# Beyond RGB: Integration of an 8‑Channel Digital Light Sensor into a Fully Automated Platform for Colorimetric Sensor Arrays

**DOI:** 10.1021/acs.analchem.5c04288

**Published:** 2025-09-11

**Authors:** Josiele Aparecida Magalhães Conrado, Diogo Morais de Jesus, Caio C. S. Machado, Yugo S. N. da Mota, Sidnei Gonçalves da Silva, João Flávio da Silveira Petruci

**Affiliations:** Institute of Chemistry, 28119 Federal University of Uberlândia (UFU), Uberlândia, Minas Gerais 38400-902, Brazil

## Abstract

Sensing arrays are a powerful tool for discriminating complex samples without the need for pretreatment steps or separation techniques. Among them, colorimetric sensor arrays rely on the color change of specific reagents upon interaction with the chemicals present in the sample. Typically, the color change of each sensing element is obtained through digital image acquisition, followed by color data extraction using a color space (i.e., RGB model). Despite its widespread application, the RGB model limits color information to only three channels, which can reduce the method’s discrimination capability. Moreover, the image acquisition, followed by color extraction, can be time-consuming, particularly when the array includes dozens of reagents. In this study, we propose, for the first time, the integration of a fully automated analytical platform with an 8-channel digital light sensor for application in colorimetric sensor arrays. As a proof-of-concept, the system was used to discriminate various food products (e.g., tea, sauces, soda, and juice) based on their acid–base properties using six pH indicators. Eleven microliters of each indicator were deposited onto rectangular filter paper, followed by the addition of each food sample. The paper sensors were then placed in their respective positions on the automated platform. Color changes were measured using a digital light sensor integrated into the system. The total data acquisition time was less than 1 min. The resulting data were processed by using principal component analysis (PCA) and hierarchical cluster analysis (HCA), successfully enabling sample discrimination. This study presents a simple, fast, low-cost, and fully automated analytical platform that enhances the capabilities of colorimetric sensor arrays by coupling them with a multichannel optical detection system.

## Graphical abstract

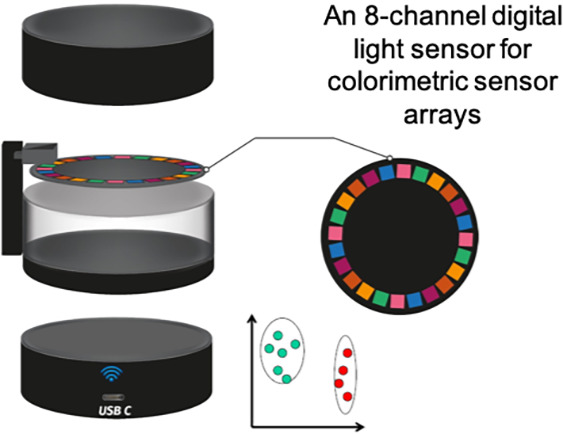

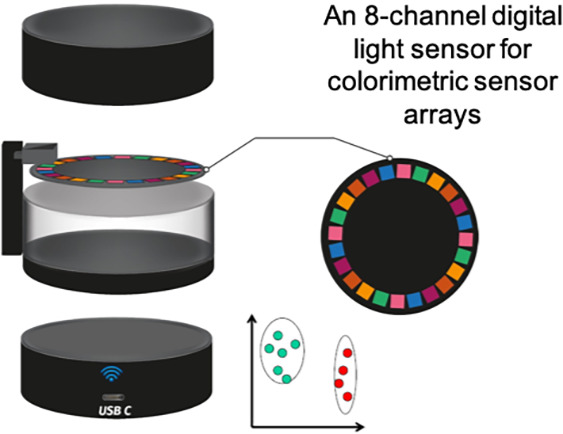

## Introduction

The colorimetric sensor array was first introduced in the early 2000s and employs cross-responsive sensor elements based on nonspecific chemical interactions for the discrimination of analytes or samples.^1^ It consists of an array of chemoresponsive dyes that generate a unique response profile upon exposure to chemical compounds.^2^ This approach features cross-reactive sensing rather than specific reagent–analyte interactions, enabling the recognition and discrimination of chemically similar target samples.^3^ Often referred to as an optoelectronic nose^4^ or optoelectronic tongue,^5^ the sensor array functions like a detective, gathering evidence from each colorimetric reaction to help reveal the identity of the analyte or mixture. The color change of each sensor element, resulting from various strong or weak chemical interactions (e.g., bond formation via Lewis acid–base interactions or van der Waals forces), is used to create a high-dimensional and distinctive response pattern for each sample.

Chemical analysis of complex samples, such as food products, is performed for various purposes including quality control, adulteration detection, degradation profiling, and contamination assessment. Component-by-component (CBC) analysis is employed when specific target compounds are directly related to the analytical goal.^6^ In such cases, separation techniques (e.g., GC, HPLC, CE) coupled with appropriate detectors (e.g., FID, DAD, MS) are considered the gold standard.^7^ However, for many other applications, a holistic approach, in which the overall response to a complex mixture is analyzed, is more appropriate. In this context, colorimetric sensor arrays represent a powerful tool. For example, 18 beer samples were successfully discriminated using a colorimetric sensor array composed of 36 hydrophobic dyes printed on hydrophobic membranes.^8^ Digital imaging of the dye spots after interaction with the liquid-phase generated a color change profile that served as a unique fingerprint for each sample. Similar strategies have been applied to discriminate rice,^9^ green teas,^10^ meats,^11,12^ oils,^2^ and various dairy products^13,14^ based on the interaction of either volatile or liquid-phase constituents with the corresponding sensing arrays.

Compact spectrometers provide full-spectrum with high resolution across the visible range due to the presence of sophisticated monochromators and optical components. However, such instruments are typically expensive and bulky and do not transmit data via Wi-Fi or Bluetooth directly to a spreadsheet; they are not designed for performing sequential colorimetric measurements directly on paper-based substrates. These limitations have led to the widespread adoption of more accessible alternatives, such as smartphones and scanners, for colorimetric analyses. Typically, the reagents are deposited onto a solid substrate and exposed to the sample, and digital images are captured before and after exposure for subsequent processing. This procedure is usually carried out with the aid of a smartphone or a flatbed scanner, followed by the extraction of RGB color information with dedicated software (e.g., ImageJ). The resulting data are represented as a vector of 3*N* dimensions, where *N* = total of sensor elements, and are then organized into spreadsheets for postprocessing and application of pattern recognition or chemometric techniques.^15^ Despite its portability and ease of use, this approach is often time-consuming, labor-intensive, and nonautomated, making it prone to variability and reduced measurement reproducibility. Additionally, the use of the RGB color model limits the analysis to only three channels, restricting the full exploitation of the visible spectrum and potentially reducing the method’s discrimination power.

These limitations can be overcome by combining mechanization and automation within an analytical platform, thereby enhancing the analytical capabilities of the colorimetric sensor array approach. Mechanization in colorimetric measurements refers to the use of devices that replace or assist human effort in specific tasks, such as sample positioning or ensuring consistent lighting. Automation, on the other hand, integrates multiple processes to perform tasks autonomously. A miniaturized system that is low-power and low-cost and yet offers high stability and reproducibility is highly desirable in the design of novel analytical platforms. Hand-held optoelectronic and cell-phone microplate readers were previously demonstrated in promising applications as easy-to-use imaging tools to acquire colorimetric data.^16,17^ However, both are based on cell-phone cameras and are in the RGB color model.

In this scenario, digital color sensors—such advanced multichannel devices like the AS7262 and AS7341—have emerged as attractive alternatives to image-based acquisition systems that require software for converting captured images into analytical signals, as is the case with scanners and smartphone cameras.^18,19^ These sensors offer the advantage of directly acquiring spectral information through built-in photodetectors and integrated optical filters, eliminating the need for laborious image processing steps.^20^ In particular, AS7262 provides six discrete spectral channels across the visible range, while AS7341 expands this capability with eight channels in the visible range and one additional near-infrared channel, thereby delivering enhanced spectral resolution. This improved resolution contributes to greater selectivity and more effective color discrimination, which are crucial for reliable colorimetric analysis. Furthermore, both sensors operate with 16-bit analog-to-digital converters, in contrast with the 8-bit-based RGB model, allowing for higher resolution in signal acquisition. Besides, this sort of sensors enables all-in-one integration with microcontrollers (e.g., Arduino or ESP32) for real-time data transmission via Wi-Fi, Bluetooth, or other wireless communication protocols directly to a PC, laptop, or smartphone.

Recently, several applications have demonstrated the use of digital color sensors for the development of analytical methods.^21–24^ An automated and mechanized analytical platform was recently developed for quantitative colorimetric analysis, employing the Berthelot reaction for the selective determination of ammonium in aqueous samples.^25^ However, the integration of digital color sensors with mechanization and automation in the context of a colorimetric sensor array approach has not yet been reported. In this study, we propose an analytical platform that combines an 8-channel digital light sensor with a 3D-printed rotating sample wheel containing 24 measurement positions, offering a mechanized and automated alternative to conventional colorimetric sensor array methods. Six reagents (i.e., pH indicators) were immobilized on paper-based devices, and the resulting color changes, arising from interactions between the reagents and the sample, were used to generate a unique fingerprint profile for each product. Using chemometric tools such as principal component analysis (PCA) and hierarchical clustering analysis (HCA), we demonstrate, as a proof-of-concept, the effective discrimination of 11 food products based on their acid–base characteristics.

## Experimental Section

### Chemicals and Solutions

Thymol blue, bromocresol green, bromocresol purple, methyl red, phenol red, and epsilon blue were obtained from Synth (Brazil). Working solutions were prepared by dissolving 10 mg of each dye in 10 mL of ethanol or an ethanol/water mixture (60:40, v/v). Deionized water was obtained using a Milli-Q system (Millipore, USA), and ethanol was purchased from Qhemis (São Paulo, Brazil).

Lactic acid, diphenylamine, triethylamine, and formic acid were obtained from Sigma-Aldrich (St. Louis, Missouri, USA). Acetic acid and ammonium hydroxide were purchased from Dinâmica (Brazil). Solutions of each analyte were prepared by dilution to a final concentration of 1 mmol L^–1^.

### Samples Preparation

As a proof-of-concept, the potential of the multichannel-based colorimetric sensor array to distinguish between food samples with distinct acid–base behavior was evaluated using various dairy and nondairy products. Sparkling water, Brazilian sugar cane spirit, lemon soda, lychee juice, vinegar, and soy sauce were purchased from a local supermarket (Uberlândia, MG, Brazil) and used as received. Egg white samples were manually separated from whole eggs.

Green and chamomile teas were prepared according to the instructions on the packaging: 200 mL of boiling water was added to a beaker containing the respective tea bag. After 10 min of infusion, the tea was allowed to cool at room temperature before analysis. Rosemary and basil teas were prepared using fresh plant leaves. In each case, 150 mL of boiling water was added to 0.1 g of the plant material and allowed to infuse for 10 min. The resulting solutions were then filtered using quantitative filter paper and allowed to cool to room temperature prior to analysis.

### Automated Analytical Platform for Colorimetric Measurements

The concept of the automated analytical platform employed in this study was fully described in previous work^25^ and illustrated in Figure 1. The Smart-PAD platform is based on an 8-channel digital light sensor (AS7341, Adafruit, USA) positioned above a sample wheel containing 24 measurement spots. Each spot is symmetrically arranged around the wheel and accommodates a square paper-based device with dimensions of 10 mm × 10 mm. A stepper motor rotates the sample wheel, enabling precise alignment of each sample with the light sensor and allowing light reflectance measurements to be performed.

**Figure 1:**
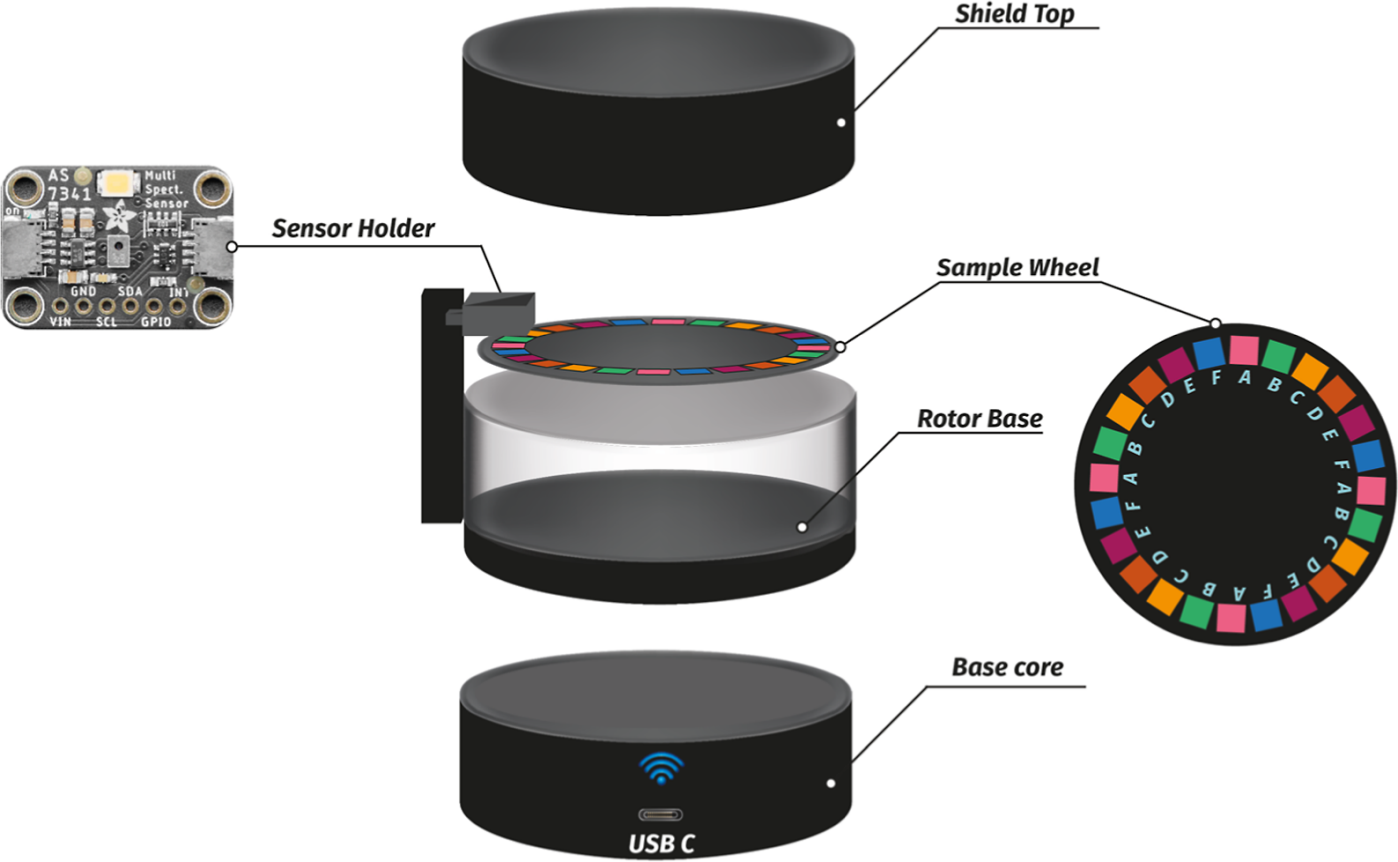
3D model of the automated platform configurated for the colorimetric sensor array approach.

All the electronic components are operated with the aid of an ESP32 microcontroller, with data transmitted wirelessly to a Raspberry Pi 3-based analysis hub using Wi-Fi. A web-based app developed in JavaScript serves as the control interface for the analytical platform. The system architecture allows users to configure measurement parameters such as detector gain, LED intensity, number of samples, and measurement mode. Upon pressing “Start Measurements,” the sample wheel is automatically positioned and the light intensity that reaches the detector is acquired. Moreover, the platform can be powered by a portable 10,000 mAh battery, providing up to 10 h of autonomy and enabling field-deployable operation.

The 8-channel digital light sensor contains integrated filters, allowing for discrete measurements across the visible spectrum at 415, 445, 480, 515, 555, 580, 630, and 680 nm. Results are expressed as the total light intensity (LUX) for each channel. Data from the analysis are automatically organized into spreadsheets for subsequent processing.

### Preparation of the Paper-Based Colorimetric Sensor Array and Analytical Procedure

Whatman filter paper No. 1 (Sigma-Aldrich, USA) was used to fabricate the paper-based analytical devices employed for dye impregnation. Square PADs measuring 10 × 10 mm were designed by Inkscape and produced by using a commercially available ScanNCut printer (Brother SDX85, Japan), equipped with an integrated scanner and automated cutting functions. Prior to use, the paper pads were stirred in a 1:1 ethanol/water solution for 30 min and then dried at room temperature for 12 h to ensure complete solvent evaporation.^26^

After preparation, the papers were positioned in their respective locations on the sample wheel platform. The colorimetric sensor array consisted of six different pH indicators applied in the following sequence: thymol blue (A), bromocresol green (B), bromocresol purple (C), methyl red (D), phenol red (E), and epsilon blue (F).

An aliquot of 11 μL of each indicator solution was manually deposited onto the central area of the respective paper-based device and allowed to dry for 10 min. Although this step was performed manually in this study, we emphasize that this procedure is fully compatible with automation. In fact, a 3D-printed liquid handling robot specifically designed for precise and reproducible dispensing was recently published,^27^ which can be readily integrated into the current workflow. The light intensity of each spot was then measured, stored, and labeled as the light intensity at each specific wavelength before interaction with the sample (e.g., *I*_B415nm_). Subsequently, 11 μL of the sample was added to each indicator-containing PAD, followed by a 10 min drying time. After this period, the light intensity was measured again, stored, and labeled as the light intensity after exposure to the sample (e.g., *I*_A415nm_). The final response signal for each sensor element (*R*_n_) was calculated as the subtraction of the raw light intensity (eq 1) or the absorbance (−log *I*/*I*_0_) values (eq 2). It is important to note that *I*_0_ refers to the sensor response of the blank paper after addition of the solvent or sample. Additionally, for raw intensity measurements, both *I*_A_ and *I*_B_ signals were corrected by subtracting the response of the blank paper after the addition of the solvent and sample, in order to minimize interference from the color of the solutions.

1 $$R_{n}=I_{A}-I_{B}$$

2 $$R_{n}={ABS}_{A}-{ABS}_{B}$$

## Results and Discussion

### Effect of Paper Moisture on the Light Intensity Signal

The AS7341 multispectral sensor offers notable advantages for colorimetric measurements when compared with traditional imaging devices such as scanners or smartphone cameras. While these conventional systems rely on the RGB model, which captures color information using only three broad channels (red, green, and blue), AS7341 features eight discrete spectral channels in the visible range (415–680 nm). This improved spectral resolution enhances the sensor’s ability to distinguish between subtle variations in color, leading to more effective separation of overlapping spectral regions and better analytical performance in complex samples. Additionally, AS7341 operates with 16-bit resolution, in contrast to the 8-bit depth typical of RGB-based devices. An inherent limitation of a digital light sensor equipped with a 16-bit analog-to-digital converter (ADC) is detector saturation. To prevent this behavior, the optimal LED current and sensor gain settings for the AS7341 sensor were previously optimized for colorimetric measurements in both liquid and solid (i.e., paper-based) samples.^19^ Table S1 presents the optimal conditions for each sensor channel. This higher dynamic range enables the detection of finer differences in color intensity, which is especially valuable in applications requiring the precise quantification of chromatic changes. To the best of our knowledge, this is the first report of AS7341 being applied in this context, providing a promising alternative for the development of low-cost, high-resolution colorimetric sensing systems in analytical chemistry. Due to the design of the analytical platform, light detection is performed in the reflectance mode. The light emitted by the LED embedded in the detector is reflected by the paper surface and returns to the detector. If any portion of the light is absorbed by the dye impregnated onto the paper surface, then the intensity of the reflected light decreases, leading to a lower signal detected by the digital light sensor.

In such cases, the effect of light scattering caused by the solvent presented in the PAD is not negligible and may result in a pronounced variation in the light intensity acquired by the detector. To evaluate the effect of paper moisture in the light intensity signal, 11 μL of bromocresol green and methyl red were added to three papers, respectively, and the light intensity of each channel was recorded for 16 min. As can be seen in Figure S1a,b, the analytical signals for all channels are affected by the paper moisture. A stable reading was only reached when the paper was dry, indicating that the measurement should be carried out after 10 min.

### Evaluation of the 8-Channel Digital Light Sensor as Detector for Colorimetric Sensor Array

Initially, the robustness of the platform in providing precise signals was evaluated by measuring the light intensity (i.e., reflectance) of paper-based devices impregnated with sunset yellow dye. Herein, PADs were placed in each of the 24 positions and the light intensity was recorded. A relative standard deviation of 2.2% was obtained, indicating minimal variation in the acquired signal across all positions.

As mentioned in the Introduction section, colorimetric sensor arrays rely on changes in dye color before and after the interaction with the sample. Color information is typically extracted from digital images using the RGB model, a three-dimensional color space based on the red, green, and blue channels. In this study, we investigate the potential of an 8-channel digital light sensor as a detector for a colorimetric sensor array. The AS7351 digital sensor measures light intensity across the visible spectrum, providing discrete readings at 415 (±29), 445 (±33), 480 (±36), 515 (±40), 555 (±42), 580 (±44), 630 (±53), and 680 (±60) nm. Although not all channels are relevant for a given dye, a single color can be monitored across multiple wavelengths, in contrast with the RGB model. For example, the blue color may be detected at 415, 445, and 480 nm. This multispectral approach may enhance the discriminative power of the colorimetric sensor array.

Solutions of lactic acid (p*K*_a_ = 3.8), formic acid (p*K*_a_ = 3.7), acetic acid (p*K*_a_ = 4.7), citric acid (p*K*_a_ = 3.7, 4.7, and 6.4), diphenylamine (p*K*_b_ = 12.2), triethylamine (p*K*_b_ = 3.2), and ammonium hydroxide (p*K*_b_ = 4.7), each at a concentration of 1 mmol L^–1^, were used to evaluate the discriminatory potential of the proposed approach. Paper-based analytical devices containing six pH indicators were prepared as described in the Experimental Section, and the light intensity for each channel was recorded.

Subsequently, 11 μL of each analyte solution was pipetted onto the colorimetric sensor array and light intensity measurements were performed. Classification of the acidic and basic species based on the high-dimensional response profile of the 8-channel colorimetric sensor array was carried out using principal component analysis (PCA), an independent unsupervised technique. The used data set was generated from replicate measurements, using two response formats based on the subtraction of (i) raw intensity (LUX) and (ii) absorbance values (−log *I*/*I*_0_). All PCAs and HCAs were calculated through mean-centered data using the PLS toolbox under the MATLAB environment.

The two-dimensional PCA score plots for both data sets are shown in Figure 2a and b, accounting for 80.75% and 75.03% of the total explained variance for raw light intensity and absorbance differences, respectively. In general, RGB data sets obtained from colorimetric sensor arrays are considered high-dimensional and thus typically require more than two or three components to capture over 95% of the total discrimination information, as previously reported in the literature.^8^ Based on the analysis of PC1 and PC2, distinct clusters were formed, grouping compounds according to their acid–base behavior. Ammonium hydroxide and triethylamine were completely separated from the other compounds along PC1, while three additional groups, one containing diphenylamine, deionized water, and acetic acid and another comprising citric acid, lactic acid, and formic acid, were formed along PC2 (Figure 3). These classifications align with the p*K*_a_ values of the respective compounds. Among the clusters, only citric acid, lactic acid, and formic acid were not clearly separated in the PC1 vs PC2 space. However, by including PC3, the separation of citric acid from lactic and formic acid was achieved (Figures S2 and S3). Moreover, when the results obtained using raw light intensity and absorbance were compared, no significant difference in the discriminatory power of the sensor array was observed. Therefore, the raw intensity was selected due to its inherent simplicity and ease of use.

**Figure 2:**
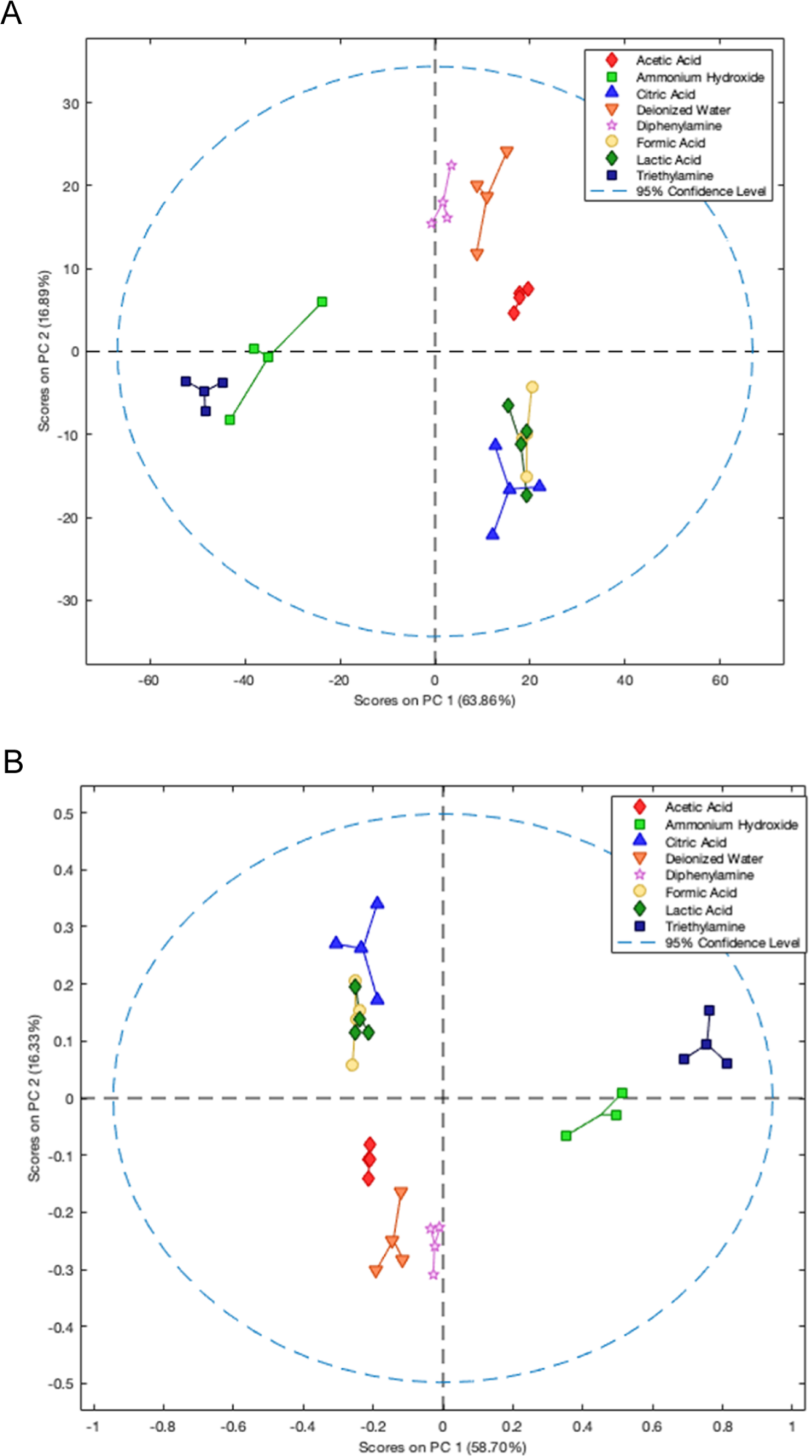
PCA score plots showing two-dimensional separation of acid and basic compounds using two response formats based (a) raw intensity (LUX) and (b) absorbance values (−log *I*/*I*_0_).

**Figure 3:**
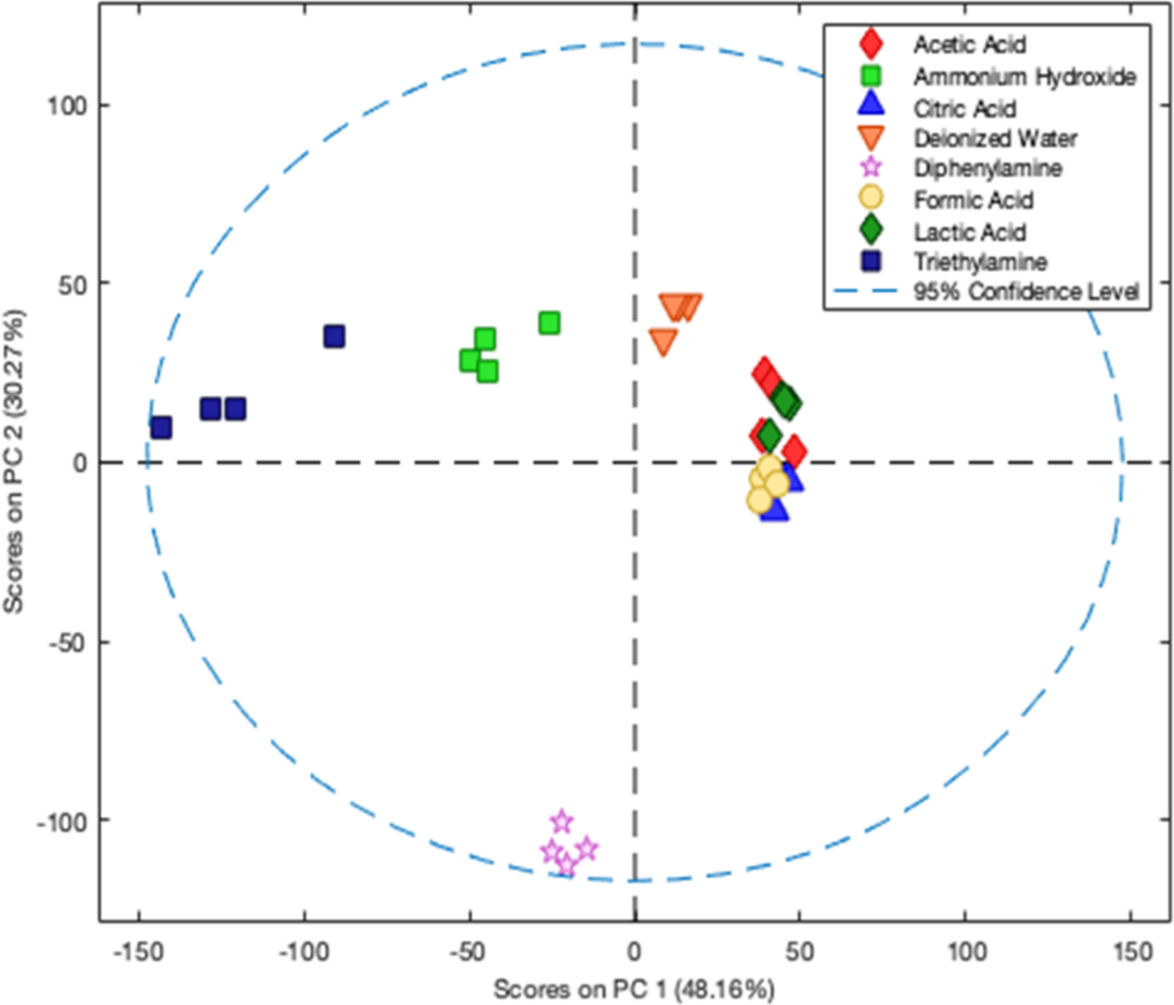
PCA score plots showing two-dimensional separation of acid and basic species using the RGB color space as input data for each colorimetric sensor.

Additionally, the loading plots of the first two principal components allow the identification of which pH indicators have a more pronounced influence on the classification of acid–base compounds. As shown in Figure S3, the signals associated with the dyes bromocresol blue and thymol blue, with light intensity measured at 445, 515, 555, and 590 nm, were the most critical for enhancing the separation of the analytes. It is important to note, however, that all other dyes included in the array also contribute to the classification performance by increasing the dimensionality of the data set.

Furthermore, the capability of the 8-channel digital light sensor as a detector for the colorimetric sensor array was demonstrated by the successful separation of acid–base species. Next, we compared the performance of this approach with the conventional “gold standard” color model, also known as the RGB color space. For this comparison, digital images of the colorimetric sensor array were acquired by using a flatbed scanner. The response signal was defined as the difference in RGB values before and after the interaction between the dyes and the analytes. The PCA score plot of PC1 versus PC2 using this data set is shown in Figure 4 and accounts for 78.43% of the total explained variance. Similarly to the previous approach, distinct clusters were formed for ammonium hydroxide, triethylamine, and diphenylamine (along PC1) and for deionized water and the acids (along PC2). However, this model was not capable of discriminating between the acid species, which remained grouped within a single cluster. These results demonstrate that the higher-dimensional response provided by the 8-channel light sensor enhances the discriminatory power of the colorimetric sensor array, enabling the differentiation of chemically similar species.

**Figure 4:**
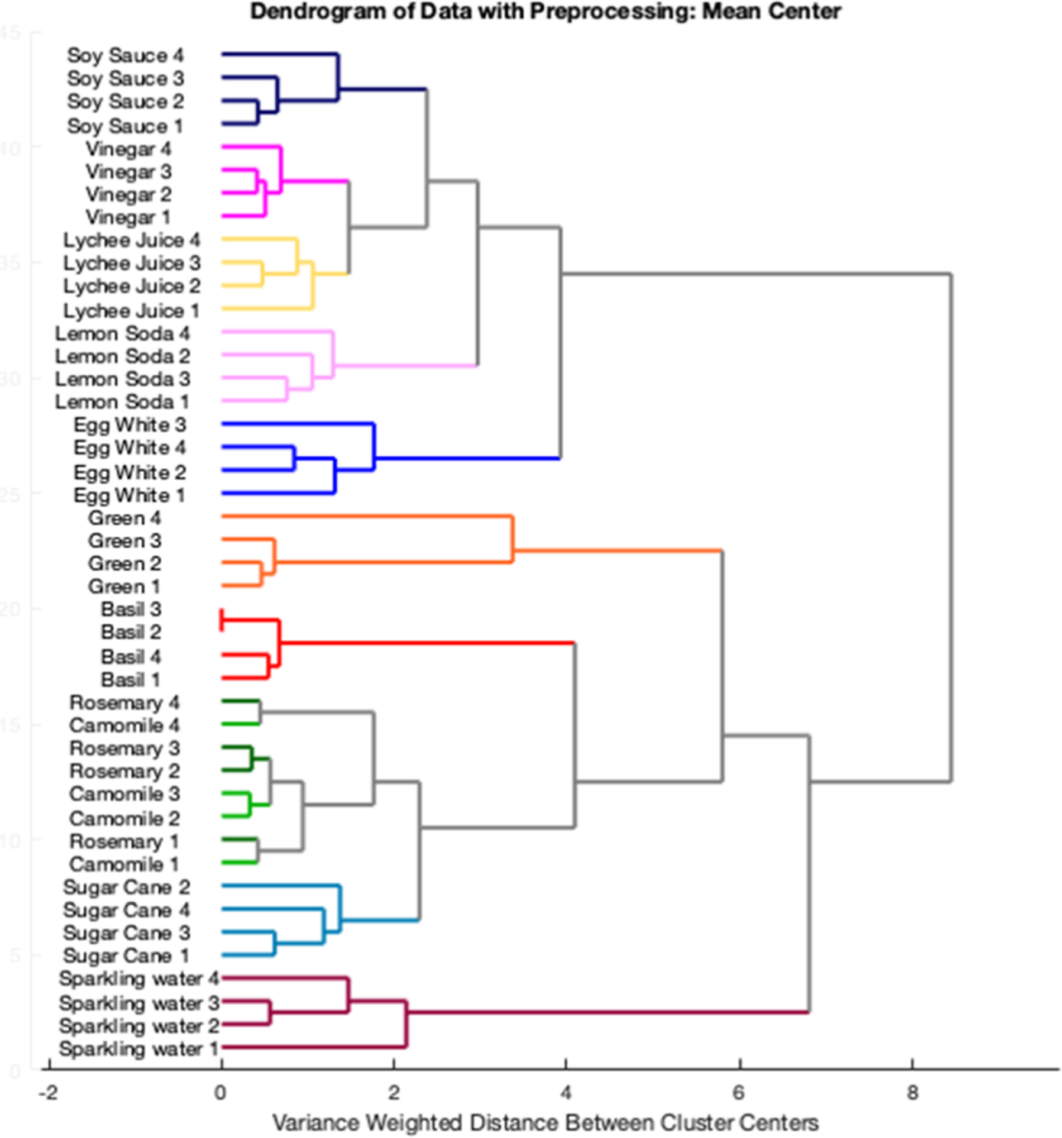
HCA dendrogram of the 8-channel colorimetric sensor array response to different food samples using minimum variance (Ward́s method).

### Application of the Automated and 8-Channel Light Sensor-Based Platform for the Discrimination of Food Samples

Next, the applicability of the proposed automated platform based on the combination of an 8-channel digital light sensor with the colorimetric sensor array strategy was evaluated for the discrimination of food samples with different acid–base behaviors. Sparkling water (pH = 5.5), Brazilian sugar cane spirit (pH = 3.7), lemon soda (pH = 3.1), lychee juice (pH = 3.5), vinegar (pH = 3.1), soy sauce (pH = 3), egg white (pH = 9), and green (pH = 5.6), chamomile (pH = 5.8), rosemary (pH = 5.8), and basil (pH = 5.3) teas were used as proof-of-concept. All of the samples were prepared as indicated in the Experimental Section. After the analytical procedure, the final response of each sensor element was considered the subtraction of the light intensity from before and after contact between the dye and the sample.

With the generated data set, two different independent unsupervised techniques were employed: PCA and HCA (Hierarchical Clustering Analysis). HCA is an agglomerative method to establish a hierarchy between samples, clustering them according to their similarity among the data collected. The results revealed the clustering of each type of food sample into distinct branches, which was made using the simple Euclidean distance with mean-centered data. The overall total response of the array is a very comparative demonstration of the availability of this approach as a fingerprint method. The HCA results—abridged as a dendrogram displayed in Figure 4—show ten well-defined clusters belonging to their corresponding food sample. Among them, only rosemary and chamomile teas were classified as the same cluster. It can be explained by the fact that both have the same pH (5.8).

Next, PCA was employed to evaluate the discrimination power of the array. The combination of the three first principal components (PC1, PC2, and PC3) accounted for 65.52% of the total explained variance. Considering the analysis of only the first two most significant principal components (Figure 5), it can be observed that egg white, lemon soda, lychee juice, and soy sauce were separated from sparking water, Brazilian sugar cane, and the teas by PC1. Additionally, the majority of the samples were grouped in distinct clusters, enabling their classification, with exception of Brazilian sugar cane and rosemary and chamomile teas, that belonged to the same cluster. By including PC3, the Brazilian sugar cane was successfully separated from the other teas. All the results obtained in the descriptive-qualitative techniques revealed the high capability of the array sensing strategy to discriminate similar food samples, thus increasing the selectivity of colorimetric methods that can be employed in a variety of real-world scenarios.

**Figure 5:**
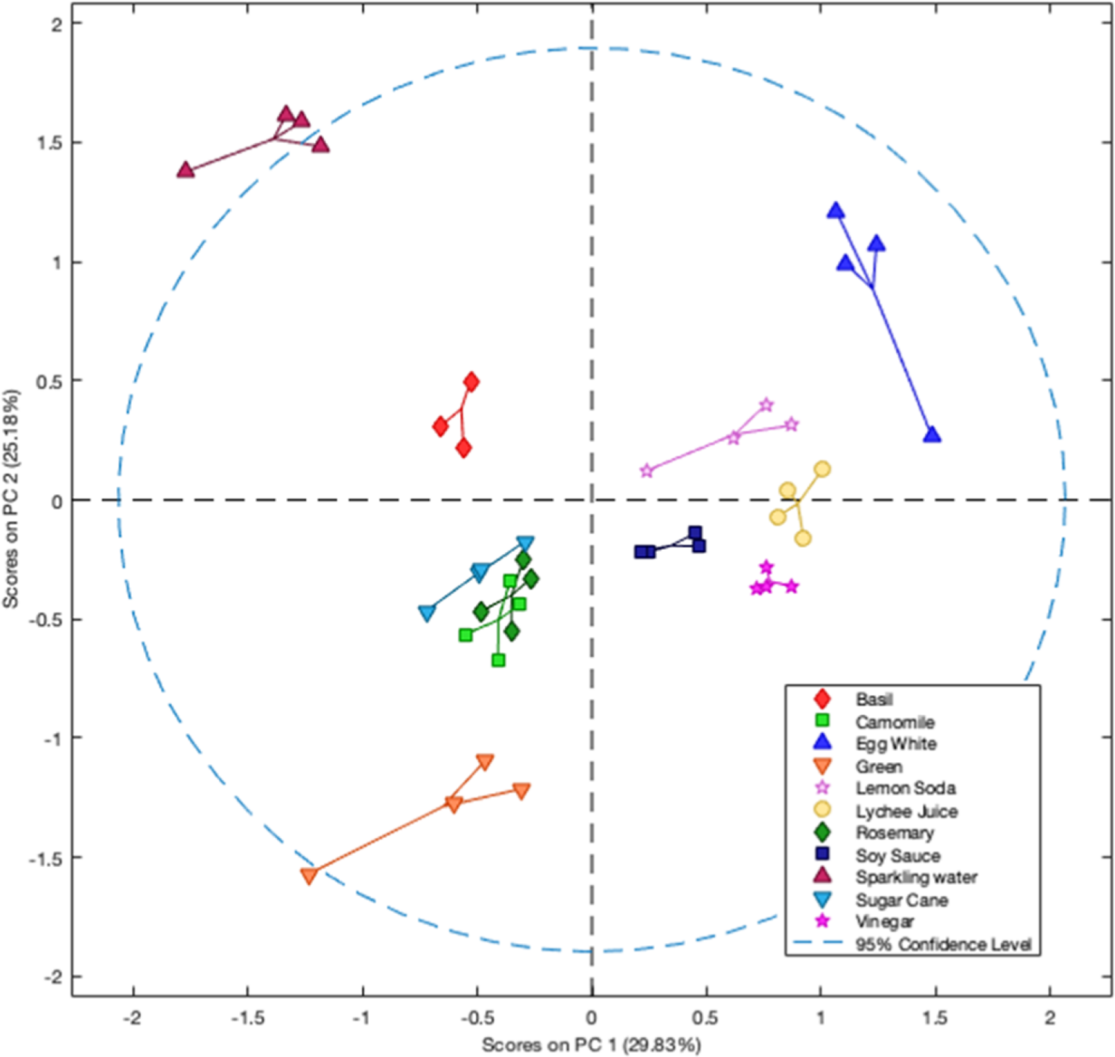
PCA score plot showing two-dimensional separation food samples based on the 8-channel digital light sensor and their acid–base behavior.

### Investigation on the Influence of Each Dye and Wavelength Channel on the Classification of Samples with Different Acid–Base Behavior

The principle of acid–base indicators is based on the equilibrium between a weak acid (HIn) and its conjugate base (In^–^). Depending on the pH of the solution, one of these species will predominate. The ratio between the concentrations of the weak acid and its conjugate base ([HIn]/[In^–^]) determines the predominant color of the solution containing the indicator. As a rule of thumb, if this ratio is greater than or equal to 10, the color associated with the acidic form will predominate; if it is less than 0.1, the color of the basic form will prevail. The range between these two conditions is known as the indicator’s transition range, where the observed color is a mixture of the acidic and basic forms. One major advantage of using a multichannel digital light sensor is its ability to monitor the entire color transition of the dyes across the visible spectrum.

By taking advantage of principal component analysis, it is possible to identify which pH indicators and their corresponding wavelengths have the most significant impact on differentiating the food samples. The Variable Importance in Projection (VIP) scores for the two principal components were summed, along with the final analytical response for each channel. Table 1 presents the ranking of dyes based on their importance in sample classification, along with their most relevant wavelength channels.

**Table 1:** Influence of Each pH Indicator in the Classification of the Food Samples and Their Respective Most Active Wavelength Channels

importance	pH indicator	wavelength channels
1	bromocresol green	445, 590, and 630 nm
2	thymol blue	445, 515, and 555 nm
3	phenol red	445, 480, and 515 nm
4	bromocresol purple	445, 480, and 680 nm
5	epsilon blue	445 and 555 nm
6	methyl red	515, 555, and 590 nm

By comparing the absorbance spectrum of each pH indicator with the data presented in Table 1, it is evident that the relevant wavelength channels monitored by the AS7341 align with the absorbance bands of each indicator, in either its acidic or basic form. Figure 6 illustrates the correspondence between the digital light sensor channels and the absorbance spectrum of methyl red, as an example. This confirms the capability of the 8-channel digital light sensor to monitor the color changes of pH indicators under acidic or basic conditions, before and after exposure to the samples. The broad operational range of the multichannel light sensor represents a significant alternative for colorimetric measurements in sensor array-based approaches.

**Figure 6:**
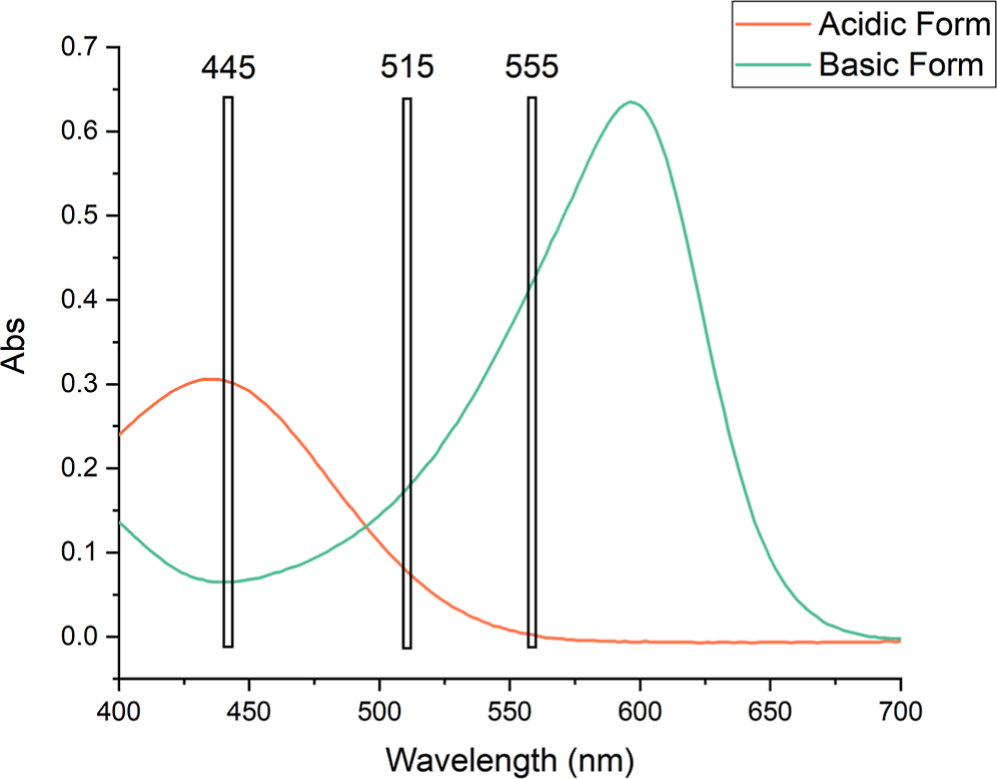
Absorbance spectra of methyl red in acidic and basic forms, with the respective wavelength channels monitored by the AS7341 digital light sensor.

## Conclusions

In this study, we proposed the use of an 8-channel digital light sensor as a detector for the colorimetric sensor array approach, replacing the conventionally used RGB color space. In addition to expanding the number of response channels from three to eight, the miniaturized light sensor can be operated via a microcontroller, thereby increasing the degree of automation. By integrating the digital light sensor with simple electronic components (e.g., stepper motor) and 3D printing, we developed a fully automated analytical platform capable of performing sequential colorimetric measurements and wireless data transmission. Using this approach, six pH indicators were immobilized onto paper-based analytical devices, and the multidimensional signals obtained before and after interaction with the samples were used to discriminate between acid and base species, as well as among 11 food samples.

Principal component analysis was applied to data sets generated both from the digital light sensor and the RGB color space to evaluate the discrimination power of each method. The results showed that both approaches produced distinct clusters for each analyte; however, the 8-channel sensor was more effective in differentiating closely related species, such as acids with similar p*K*_a_ values. This suggests that our approach enhances the discriminatory capacity of colorimetric sensor arrays by providing higher-resolution color information compared with the RGB model. Moreover, we demonstrated that digital light sensors can be integrated with microcontrollers and automated platforms to enable rapid, sequential signal acquisition, typically within 1 min. For multivariate strategies, such as colorimetric sensor arrays, this represents a significant improvement in signal acquisition time, portability, data processing, automation, ease of use, and mechanization. Although the proposed platform was evaluated using simple pH indicators (acid–base chemistry), it is compatible with any configuration of a colorimetric sensor array, including solvatochromic, vapochromic, and redox dyes, Lewis basic dyes (e.g., metalloporphyrins), chromogenic aggregative indicators (e.g., AuNPs and QDs), and even fluorescent dyes. Therefore, other analytes and more complex sample differentiations can be achieved by tailoring the array configuration accordingly. By offering an alternative to traditional RGB-based color extraction, we anticipate the application of this approach in a wide range of analytical scenarios. Additionally, a recently introduced 12-channel digital light sensor presents a promising alternative for future developments in the field of colorimetric sensor arrays.

## Supplementary Material



## Notes

Additional results, such as PCA score plots, and the raw data set obtained by each individual sensor element (PDF)

## References

[1] Suslick K. S., Rakow N. A. (2000). A Colorimetric Sensor Array for Odour Visualization. Nature.

[2] M.
Conrado J. A., Sequinel R., Dias B. C., Silvestre M., Batista A. D., Petruci J. F. D. S. (2021). Chemical QR Code: A Simple and Disposable
Paper-Based Optoelectronic Nose for the Identification of Olive Oil
Odor. Food Chem..

[3] Li Z., Suslick K. S. (2021). The Optoelectronic
Nose. Acc.
Chem. Res..

[4] Pinheiro N. D., Freire R. T., Conrado J. A. M., Batista A. D., da Silveira
Petruci J. F. (2021). Paper-Based Optoelectronic Nose for Identification
of Indoor Air Pollution Caused by 3D Printing Thermoplastic Filaments. Anal. Chim. Acta.

[5] Dias B. C., Batista A. D., da Silveira
Petruci J.
F. (2021). μOPTO: A Microfluidic
Paper-Based Optoelectronic Tongue as Presumptive Tests for the Discrimination
of Alkaloid Drugs for Forensic Purposes. Anal.
Chim. Acta.

[6] Suslick K. S., Bailey D. P., Ingison C. K., Janzen M., Kosal M. E., McNamara W. B., Rakow N. A., Sen A., Weaver J. J., Wilson J. B., Zhang C., Nakagaki S. (2007). Quim. Nova.

[7] Fraga-Corral M., Carpena M., Garcia-Oliveira P., Pereira A. G., Prieto M. A., Simal-Gandara J. (2022). Analytical
Metabolomics and Applications in Health,
Environmental and Food Science. Crit. Rev. Anal.
Chem..

[8] Zhang C., Bailey D. P., Suslick K. S. (2006). Colorimetric
Sensor Arrays for the
Analysis of Beers: A Feasibility Study. J. Agric.
Food Chem..

[9] Arslan M., Zareef M., Tahir H. E., Guo Z., Rakha A., Xuetao H., Shi J., Zhihua L., Xiaobo Z., Khan M. R. (2022). Discrimination of Rice Varieties
Using Smartphone-Based
Colorimetric Sensor Arrays and Gas Chromatography Techniques. Food Chem..

[10] Gomes J. S., de Sousa R. M. F., Petruci J. F. da S. (2022). Paper-Based
Colorimetric Sensor Array
for the Rapid and on-Site Discrimination of Green Tea Samples Based
on the Flavonoid Composition. Anal. Methods.

[11] Li Z., Suslick K. S. (2016). Portable Optoelectronic
Nose for Monitoring Meat Freshness. ACS Sens..

[12] Salinas Y., Ros-Lis J. V., Vivancos J. L., Martínez-Máñez R., Marcos M. D., Aucejo S., Herranz N., Lorente I. (2012). Monitoring
of Chicken Meat Freshness by Means of a Colorimetric Sensor Array. Analyst.

[13] Andre R. S., Mercante L. A., Facure M. H. M., Sanfelice R. C., Fugikawa-Santos L., Swager T. M., Correa D. S. (2022). Recent Progress
in Amine Gas Sensors for Food Quality Monitoring: Novel Architectures
for Sensing Materials and Systems. ACS Sens..

[14] Kim S. Y., Li J., Lim N. R., Kang B. S., Park H. J. (2016). Prediction of Warmed-over
Flavour Development in Cooked Chicken by Colorimetric Sensor Array. Food Chem..

[15] Li Z., Askim J. R., Suslick K. S. (2019). The Optoelectronic
Nose: Colorimetric
and Fluorometric Sensor Arrays. Chem. Rev..

[16] Li Z., Suslick K. S. (2018). A Hand-Held Optoelectronic
Nose for the Identification
of Liquors. ACS Sens..

[17] Berg B., Cortazar B., Tseng D., Ozkan H., Feng S., Wei Q., Chan R. Y. L., Burbano J., Farooqui Q., Lewinski M., Di Carlo D., Garner O. B., Ozcan A. (2015). Cellphone-Based Hand-Held
Microplate Reader for Point-of-Care Testing of Enzyme-Linked Immunosorbent
Assays. ACS Nano.

[18] Cadeado A., Machado C., Oliveira G., e Silva D., Muñoz R., Silva S. (2022). Internet of Things as a Tool for Sustainable Analytical Chemistry:
A Review. J. Braz. Chem. Soc..

[19] Machado C. C. S., Cadeado A. N. S., da
Mota Y. S. N., Petruci J. F. S., Silva S. G. (2024). Evaluation of the
Performance of 3D Printed (Spectro)­Photometers
Based on Multi-Channel Color Sensors for Colorimetric Determinations. Anal. Methods.

[20] de
Jesus D. M., Delaqua F., da Silveira Petruci J. F., Silva S. G. (2025). Modular “Plug-and-Play” Photometer Based
on IoT Digital Light Sensors: A DIY Approach for In Situ Analysis. ACS Omega.

[21] Cesar
Souza Machado C., da Silveira Petruci J.
F., G. Silva S. . (2021). An IoT Optical
Sensor for Photometric Determination of Oxalate in Infusions. Microchem. J..

[22] da
Silva Sousa D., Leal V. G., dos Reis G. T., da Silva S. G., Cardoso A. A., da Silveira Petruci J.
F. (2022). An Automated, Self-Powered,
and Integrated Analytical Platform for On-Line and In Situ Air Quality
Monitoring. Chemosensors.

[23] de
Carvalho Oliveira G., Machado C. C. S., Inácio D. K., Silveira Petruci J. F. d., Silva S. G. (2022). RGB Color Sensor
for Colorimetric Determinations: Evaluation and Quantitative Analysis
of Colored Liquid Samples. Talanta.

[24] Yamada K., Suzuki K., Citterio D. (2017). Text-Displaying Colorimetric
Paper-Based
Analytical Device. ACS Sens..

[25] Machado, C. S. ; da Mota, Y. S. N. ; Petruc, J. F. S. ; da Silva, S. G. Smart-PAD: A Multi-Channel Mechanized Approach for High-Throughput Paper-Based Colorimetric Analysis. Anal. Chim. Acta, 2025, in press. 10.1016/j.aca.2025.344843 41330676

[26] Ortiz-Gomez I., Ortega-Muñoz M., Marín-Sánchez A., de Orbe-Payá I., Hernandez-Mateo F., Capitan-Vallvey L. F., Santoyo-Gonzalez F., Salinas-Castillo A. (2020). A Vinyl Sulfone Clicked Carbon Dot-Engineered
Microfluidic Paper-Based Analytical Device for Fluorometric Determination
of Biothiols. Microchim. Acta.

[27] Assunção A. D., de Jesus D. M., Terra T. R., Rocha R. G., Costa J. A. M. G., da Silva L. A. J., Fernandes G. L., Dias A. G. C., Munoz R. A. A., Silva S. G., Richter E. M. (2025). 3D-Printed
Liquid Handling Robot for the Development of Automated Analytical
Methods. J. Braz. Chem. Soc..

