# Supplementary material for: Beyond RGB: Integration of an 8‑Channel Digital Light Sensor into a Fully Automated Platform for Colorimetric Sensor Arrays
Source: Anal Chem. 2025 Sep 11;97(37):20585–93. doi: 10.1021/acs.analchem.5c04288 (PMC12461675; doi:10.1021/acs.analchem.5c04288)
Supplement: Supplementary file 1 [file ac5c04288_si_001.pdf]

SUPPLEMENTARY INFORMATION

Beyond RGB: Integration of an 8-Channel Digital Light Sensor into a Fully Automated Platform for Colorimetric Sensor Arrays

Josiele Aparecida Magalhães Conrado, Diogo Morais de Jesus, Caio C. S. Machado, Yugo S. N. da Mota, Sidnei Gonçalves da Silva e João Flávio da Silveira Petruci*

Institute of Chemistry, Federal University of Uberlândia (UFU), Uberlândia-MG, 38400-902, Brazil

*corresponding author: jfpetruci@gmail.com

Table of contents:

Table S1. Optimized parameters for the AS7341 sensor channels, including gain settings and LED currents. 2

Figure S1. Effect of the paper's moisture in the analytical signal of each channel. (A) Bromocresol green and (B) Methyl red 3

Figure S2. PCA score plots showing three-dimensional separation of acid and basic compounds using two response formats based (a) raw intensity (LUX) and (b) absorbance values (−log I/I_0)..4

Figure S3. Loading plot of the first principal component of the six pH indicators-based colorimetric sensor array. 5

Table S1. Raw dataset extracted from each individual sensor element of the 8-channel digital light sensor..6

Table S1. Optimized parameters for the AS7341 sensor channels, including gain settings and LED currents.

	**415**	**445**	**480**	**515**	**555**	**580**	**630**	**680**
**Gain (x)**	512	64	32	16	16	16	16	16
**LED Current (mA)**	20	20	50	70	60	70	70	100

A

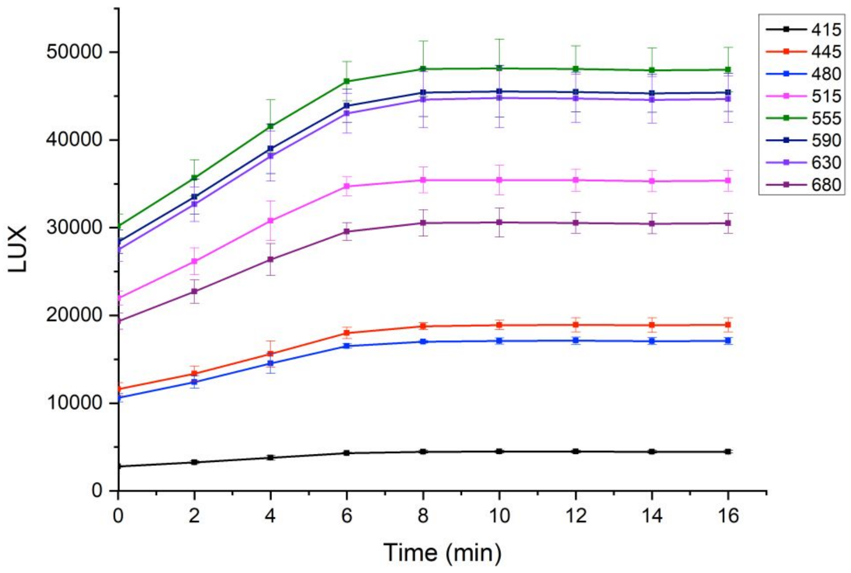

B

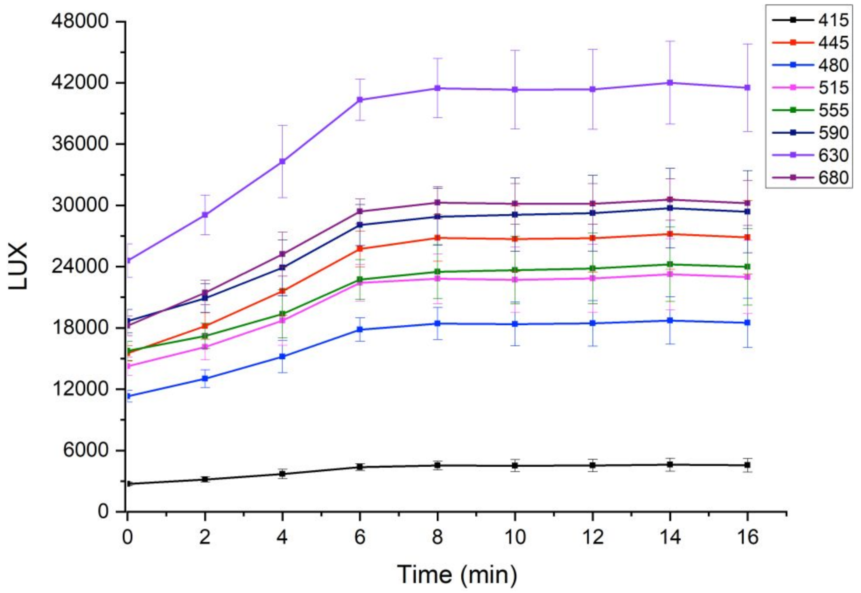

Figure S1. Effect of the paper's moisture in the analytical signal of each channel. (A) Bromocresol green and (B) Methyl red

A

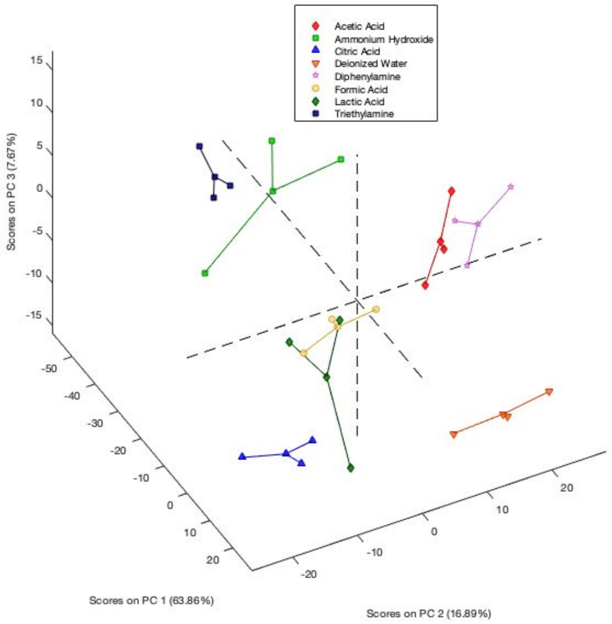

B

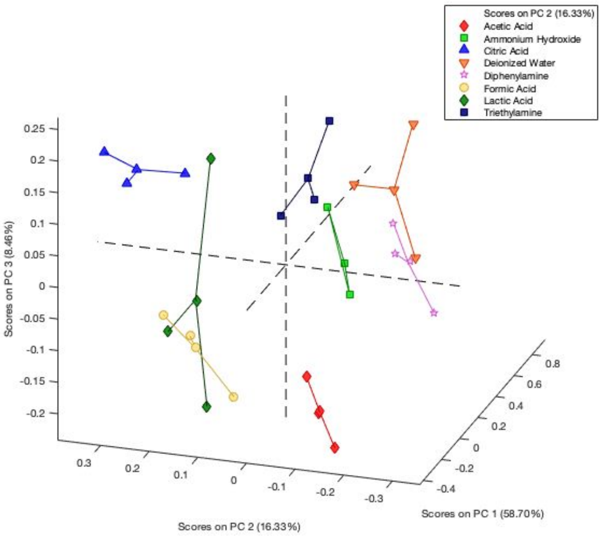

Figure S2. PCA score plots showing three-dimensional separation of acid and basic compounds using two response formats based (a) raw intensity (LUX) and (b) absorbance values (−log I/I_0).

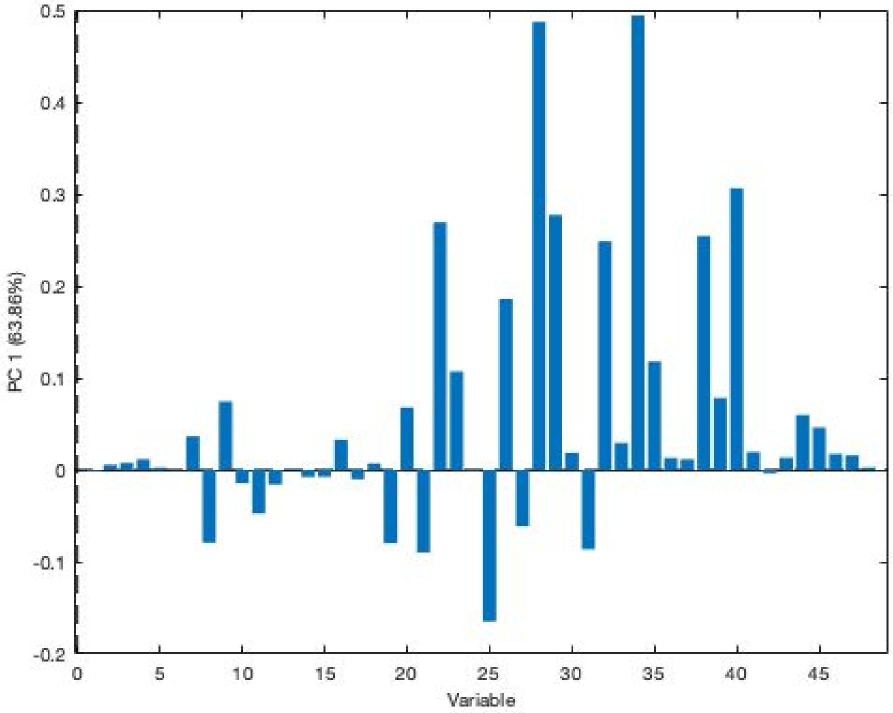

Figure S3. Loading plot of the first principal component of the six pH indicators-based colorimetric sensor array.

Table S1. Raw dataset extracted from each individual sensor element of the 8-channel digital light sensor

Analyte	B1-415	B2-415	B3-415	B4-415	B5-415	B6-415	B1-445	B2-445	B3-445	B4-445	B5-445	B6-445
Lactic Acid 1	70	20	-520	270	600	250	7810	-5910	5110	5110	4550	-60
Lactic Acid 2	520	550	580	300	270	-250	7810	-5910	5110	3100	4550	-60
Lactic Acid 3	-30	720	80	-10	600	-100	5200	-5000	1140	1870	4820	730
Lactic Acid 4	190	430	50	190	490	-30	6940	-5610	3790	3360	4640	200
Citric Acid 1	-100	-170	-160	30	100	-30	4990	-8750	-160	-160	2490	770
Citric Acid 2	-630	340	-220	-130	150	-150	2440	-6080	-60	1140	2580	140
Citric Acid 3	-460	870	-590	270	520	90	2940	-1900	-2350	3030	4050	1420
Citric Acid 4	-400	350	-320	60	260	-30	3460	-5580	-860	1340	3040	780
Acetic Acid 1	110	360	540	250	310	170	3380	-4430	4260	4260	2850	2220
Acetic Acid 2	620	480	620	190	620	270	5800	-5520	3950	1890	4360	2290
Acetic Acid 3	700	510	280	280	510	-390	6240	-4270	2130	2580	4060	-1020
Acetic Acid 4	480	450	480	240	480	20	5140	-4740	3450	2910	3760	1160
Formic Acid 1	-280	550	330	120	540	60	5410	-4820	2290	2290	5110	2110
Formic Acid 2	50	560	290	210	550	280	6210	-4090	4210	2900	5010	2050
Formic Acid 3	160	450	690	340	570	160	5120	-4670	2960	2680	5060	1910
Formic Acid 4	-20	520	440	220	550	170	5580	-4530	3150	2620	5060	2020
Deionized Water 1	440	590	-640	760	40	570	4240	-850	-20	-20	4180	540
Deionized Water 2	420	210	-20	1100	600	-210	1530	3230	-1620	5330	2790	520
Deionized Water 3	-40	830	-340	700	70	-80	3220	1210	-1740	6130	3100	1740
Deionized Water 4	270	540	-330	850	240	90	3000	1200	-1130	3810	3360	930
Triethylamine 1	370	-260	-220	-340	30	120	1780	3260	-2900	-2900	8470	3440
Triethylamine 2	-30	580	-380	-250	530	450	1780	3260	-2900	10280	8470	3440
Triethylamine 3	380	-110	-470	-350	450	-70	4890	-2990	-3080	8530	7830	970

Triethylamine 4	240	70	-360	-310	340	170	2820	1180	-2960	5300	8260	2620
Ammonium Hydroxide 1	-330	-250	-650	-820	-200	-250	-20	-3530	-4070	-4070	2730	640
Ammonium Hydroxide 2	370	360	30	-600	330	-180	4550	1340	580	5190	5500	690
Ammonium Hydroxide 3	340	840	-310	-210	760	230	3700	3050	-2390	5840	7780	2640
Ammonium Hydroxide 4	130	320	-310	-540	300	-70	2740	290	-1960	2320	5340	1320
Diphenylamine 1	10	20	650	-340	210	310	-950	-4270	4510	4510	560	1600
Diphenylamine 2	-60	70	-60	-410	220	190	-550	-4540	-300	-1930	780	970
Diphenylamine 3	140	30	50	-650	120	340	660	-5810	260	-3000	320	2080
Diphenylamine 4	30	40	210	-470	180	280	-280	-4870	1490	-140	550	1550

Analyte	B1-480	B2-480	B3-480	B4-480	B5-480	B6-480	B1-515	B2-515	B3-515	B4-515	B5-515	B6-515
Lactic Acid 1	2480	-1740	-1900	2050	3510	1520	-3690	760	-5280	2210	4260	2600
Lactic Acid 2	3730	-500	2520	2240	2620	-160	-710	8740	1620	2440	1550	-250
Lactic Acid 3	1950	-90	100	1120	3440	260	-3610	10050	-1320	-210	6440	430
Lactic Acid 4	2720	-780	240	1800	3190	540	-2670	6520	-1660	1480	4080	930
Citric Acid 1	1530	-3270	-690	620	1120	250	-5070	1750	-2850	-970	2310	350
Citric Acid 2	-260	-1340	-810	490	1190	150	-8140	5230	-3260	-1640	1430	230
Citric Acid 3	230	1120	-2130	1950	2590	620	-6830	8600	-5160	1660	6710	840
Citric Acid 4	500	-1160	-1210	1020	1630	340	-6680	5190	-3760	-320	3480	470
Acetic Acid 1	1790	-920	2360	1870	2950	680	1030	5060	2690	3020	9240	520
Acetic Acid 2	3490	-730	2310	1570	3990	1390	4850	7810	2960	2210	10660	2440
Acetic Acid 3	3880	-350	1320	1890	3620	-600	5520	7160	1440	2520	9100	-970
Acetic Acid 4	3050	-670	2000	1780	3520	490	3800	6680	2360	2580	9670	660

Formic Acid 1	900	-590	1240	1340	3070	860	-5530	7690	1440	790	3920	1480
Formic Acid 2	2080	-90	1260	2000	3250	1720	-3470	7700	1710	1060	5590	3740
Formic Acid 3	2460	-310	2320	2090	3220	1080	-2610	5440	3220	2430	5000	1910
Formic Acid 4	1810	-330	1610	1810	3180	1220	-3870	6940	2120	1430	4840	2380
Deionized Water 1	2030	1640	-1730	4220	1890	2220	3890	5400	-3600	6000	2880	3860
Deionized Water 2	2440	240	400	5820	3700	300	4910	2990	40	9080	9250	840
Deionized Water 3	570	2870	-650	3890	2270	320	1250	7130	-2130	5440	2790	750
Deionized Water 4	1680	1580	-660	4640	2620	950	3350	5170	-1900	6840	4970	1820
Triethylamine 1	2530	-1780	880	-280	2370	530	4560	-760	4480	-16240	-6210	1150
Triethylamine 2	920	2390	1290	-110	4170	1260	1290	4820	6860	-17240	-1500	2830
Triethylamine 3	3030	-1130	880	440	4280	-1630	5440	1040	5240	-14250	-320	570
Triethylamine 4	2160	-170	1020	20	3610	50	3760	1700	5530	-15910	-2680	1520
Ammonium Hydroxide 1	-60	-1590	40	-1500	1530	330	100	-100	3520	-14610	-1520	640
Ammonium Hydroxide 2	2730	1110	2160	570	3790	350	4930	3230	5550	-11580	2370	630
Ammonium Hydroxide 3	2340	2880	1080	1570	5710	1210	5030	7410	5390	-7770	6690	2110
Ammonium Hydroxide 4	1670	800	1090	210	3680	630	3350	3510	4820	-11320	2510	1130
Diphenylamine 1	-380	-640	2180	-1570	940	1780	2020	4860	1850	-2890	5820	3710
Diphenylamine 2	-310	-360	-660	-1560	950	950	880	5730	-1820	-2890	4960	1920

Diphenylamine 3	560	-1100	-620	-2710	720	1470	2850	5640	-2050	-5610	5490	2790
Diphenylamine 4	-40	-700	300	-1950	870	1400	1920	5410	-670	-3800	5420	2810

	B1-555	B2-555	B3-555	B4-555	B5-555	B6-555	B1-590	B2-590	B3-590	B4-590	B5-590	B6-590
Lactic Acid 1	-13390	6570	-6320	-350	890	660	-12190	13250	-4700	-840	-730	-650
Lactic Acid 2	-8530	19680	890	-270	-4170	-3380	-7280	22890	3130	-640	-5480	-6370
Lactic Acid 3	-11480	22400	-1430	-4120	3510	-2140	-11160	25010	-530	-4250	480	-4480
Lactic Acid 4	-11130	16220	-2290	-1580	80	-1620	-10210	20380	-700	-1910	-1910	-3830
Citric Acid 1	-12190	10480	-3610	-3270	-200	-1080	-12490	16530	-2670	-2380	-4870	-3230
Citric Acid 2	-16390	14460	-4270	-5880	-1020	-1680	-17110	19790	-3030	-5400	-3810	-3520
Citric Acid 3	-13750	17660	-6020	-1340	6310	-190	-14950	21720	-4990	-1580	-690	-1990
Citric Acid 4	-14110	14200	-4630	-3500	1700	-980	-14850	19350	-3560	-3120	-3120	-2910
Acetic Acid 1	-2390	13240	3270	770	2170	500	-3210	16230	4530	-200	-3210	-1580
Acetic Acid 2	2590	18430	3750	-150	5140	840	1170	21530	4670	-720	840	590
Acetic Acid 3	3260	16590	1840	460	3030	-3930	1380	19970	2520	-90	-640	-6050
Acetic Acid 4	1150	16090	2950	360	3450	-860	-220	19240	3910	-340	-1000	-2350
Formic Acid 1	-13260	18750	2030	-1980	1070	-990	-12730	22050	2520	-2200	-780	-2950
Formic Acid 2	-10990	16520	2180	-2820	2330	500	-10950	21430	2160	-2330	-760	930
Formic Acid 3	-9990	12920	4530	480	2460	-180	-9050	18350	5710	810	370	-1740
Formic Acid 4	-11410	16060	2910	-1440	1950	-220	-10910	20610	3460	-1240	-390	-1250
Deionized Water 1	6420	7660	-5020	3300	-4410	3370	2390	7080	-5910	2360	-5870	1810
Deionized Water 2	5830	4740	-600	6380	4350	-3050	450	4720	-1050	5320	720	-6150
Deionized Water 3	1710	8480	-3150	2450	-6000	-1680	-2630	7440	-3320	1620	-6280	-3530
Deionized Water 4	4650	6960	-2920	4040	-2020	-450	70	6410	-3430	3100	-3810	-2620
Triethylamine 1	4020	1100	2800	-32500	-22690	-2320	-1130	1500	-2600	-33200	-13200	-2990

Triethylamine 2	1120	4450	5170	-34370	-16080	2140	-3340	3200	-590	-34010	-7990	640
Triethylamine 3	4430	2780	3040	-30540	-16760	-3110	-2390	2740	-1610	-31180	-9230	-4060
Triethylamine 4	3190	2780	3670	-32470	-18510	-1100	-2290	2480	-1600	-32800	-10140	-2140
Ammonium Hydroxide 1	-350	1420	1170	-28840	-15100	-5350	-5710	1560	-3350	-29810	-10770	-7280
Ammonium Hydroxide 2	5170	3990	4330	-28770	-9320	-3520	-580	3440	1800	-29890	-5790	-4300
Ammonium Hydroxide 3	7520	8730	3810	-21440	-4540	370	1740	7480	-540	-22710	-1660	-2080
Ammonium Hydroxide 4	4110	4710	3100	-26350	-9650	-2830	-1520	4160	-700	-27470	-6070	-4550
Diphenylamine 1	8260	7900	2560	-3020	3720	1740	6200	7180	3870	-2730	1110	3910
Diphenylamine 2	4400	8540	-1640	-4120	3810	-260	2930	7570	-450	-4210	1290	1940
Diphenylamine 3	7590	10450	-1560	-7140	4110	670	4600	9520	-200	-6780	-90	2550
Diphenylamine 4	6750	8960	-210	-4760	3880	720	4580	8090	1070	-4570	770	2800

	B1-630	B2-630	B3-630	B4-630	B5-630	B6-630	B1-680	B2-680	B3-680	B4-680	B5-680	B6-680
Lactic Acid 1	-8450	15410	-4280	-850	-700	-260	-4530	3020	-2610	-540	-370	110
Lactic Acid 2	-2330	22850	4600	-740	-5380	-6690	-1180	6810	3080	-310	-3140	-3910
Lactic Acid 3	-9520	24860	-110	-4480	450	-5300	-5300	8220	-250	-2640	260	-3100
Lactic Acid 4	-6770	21040	70	-2020	-1880	-4080	-3670	6020	70	-1160	-1080	-2300
Citric Acid 1	-8060	17280	-2590	-1940	-5290	-3390	-3750	3680	-1750	-1080	-3250	-2020
Citric Acid 2	-14290	20700	-2900	-5010	-3750	-3430	-7780	5540	-1870	-3020	-2300	-2040
Citric Acid 3	-12760	22770	-5330	-1670	-2040	-1910	-6790	8010	-3290	-970	-1230	-1070
Citric Acid 4	-11700	20250	-3610	-2870	-3690	-2910	-6110	5740	-2300	-1690	-2260	-1710

Acetic Acid 1	-6180	16240	5310	0	-3850	-2250	-4320	4440	3460	-60	-2330	-1170
Acetic Acid 2	-1420	21060	5550	-570	490	830	-1380	6500	3350	-280	320	370
Acetic Acid 3	-1570	19900	2850	-20	-920	-6320	-1590	5790	1790	120	-500	-4390
Acetic Acid 4	-3060	19070	4570	-200	-1430	-2580	-2430	5580	2870	-70	-840	-1730
Formic Acid 1	-10390	21720	2640	-2230	-1010	-2910	-5630	5840	1420	-1300	-490	-1800
Formic Acid 2	-6760	22500	1600	-2390	-1110	1910	-3190	6760	1160	-1210	-570	1060
Formic Acid 3	-6280	19320	6370	560	150	-1390	-3260	5010	3630	660	210	-1220
Formic Acid 4	-7810	21180	3540	-1350	-660	-800	-4030	5870	2070	-620	-280	-650
Deionized Water 1	-4550	6920	-7250	2440	-6000	1880	-3270	2630	-4550	1320	-3430	1410
Deionized Water 2	-6360	4360	-1840	5460	480	-6550	-4370	650	-1160	3260	450	-3690
Deionized Water 3	-8730	7170	-3720	1720	-5900	-3600	-5600	3360	-2350	960	-3310	-2100
Deionized Water 4	-6550	6150	-4270	3210	-3810	-2760	-4410	2210	-2690	1850	-2100	-1460
Triethylamine 1	-5010	1410	-5150	-22180	-5620	-1640	-3270	2630	-4550	1320	-3430	1410
Triethylamine 2	-8280	3430	-4070	-22450	-860	1270	-4370	650	-1160	3260	450	-3690
Triethylamine 3	-8660	2600	-4490	-19620	-1180	-3080	-5600	3360	-2350	960	-3310	-2100
Triethylamine 4	-7320	2480	-4570	-21420	-2550	-1150	-4410	2210	-2690	1850	-2100	-1460
Ammonium Hydroxide 1	-10330	1550	-6110	-20110	-7250	-5640	-6400	-650	-3670	-8350	-4130	-2970
Ammonium Hydroxide 2	-7630	3490	80	-17000	-2530	-3760	-4950	1230	320	-6320	-1190	-2080
Ammonium Hydroxide 3	-6210	7370	-3170	-10730	330	-1280	-4480	3730	-2000	-2870	380	-570
Ammonium Hydroxide 4	-8060	4140	-3070	-15950	-3150	-3560	-5280	1440	-1780	-5850	-1650	-1870

Diphenylamine 1	-2230	6140	6080	-2870	910	5810	-2760	3260	3960	-2020	580	3690
Diphenylamine 2	-3240	6630	1190	-4510	920	3820	-2770	3380	870	-2960	540	2400
Diphenylamine 3	-3190	8680	1460	-6360	-940	3900	-2890	3880	940	-4240	-490	2390
Diphenylamine 4	-2890	7150	2910	-4580	300	4510	-2810	3510	1920	-3070	210	2830

B1: Thymol blue; B2: Bromocresol green; B3: Bromocresol purple; B4: Methyl red: B5: Phenol red; B6: Epsilon blue
